# A Novel Photocatalytic Material for Removing Microcystin-LR under Visible Light Irradiation: Degradation Characteristics and Mechanisms

**DOI:** 10.1371/journal.pone.0095798

**Published:** 2014-04-22

**Authors:** Xin Sui, Xiangrong Wang, Honghui Huang, Guotao Peng, Shoubing Wang, Zhengqiu Fan

**Affiliations:** 1 Department of Environmental Science & Engineering, Fudan University, Shanghai, China; 2 South China Sea Fisheries Research Institute, Chinese Academy of Fishery Sciences, Guangzhou, China; Tsinghua University, China

## Abstract

**Background and Purpose:**

Microcystin-LR (MC-LR), a common toxic species in contaminated aquatic systems, persists for long periods because of its cyclic structure. Ag_3_PO_4_ is an environment-friendly photocatalyst with relatively good degradation capacity for hazardous organic pollutants. This study aimed to investigate the degradation capacity of Ag_3_PO_4_ for MC-LR under visible light.

**Methods:**

An Ag_3_PO_4_ photocatalyst was synthesized by the ion-exchange method and characterized by X-ray diffraction, field-emission scanning electron microscope, and UV-Vis spectrophotometer. MC-LR was quantified in each sample through high-performance liquid chromatograph. The degradation efficiency of MC-LR was affected by initial pH, initial Ag_3_PO_4_ concentration, initial MC-LR concentration, and recycle experiments. The degradation intermediates of MC-LR were examined by liquid chromatography-mass spectrometry (LC/MS).

**Results:**

The degradation process can be well fitted with the pseudo-first-order kinetic model. The maximum MC-LR degradation rate of 99.98% can be obtained within 5 h under the following optimum conditions: pH of 5.01, Ag_3_PO_4_ concentration of 26.67 g/L, and MC-LR concentration of 9.06 mg/L. Nine intermediates were detected and analyzed by LC/MS. Three main degradation pathways were proposed based on the molecular weight of the intermediates and the reaction mechanism: (1) hydroxylation on the aromatic ring of Adda, (2) hydroxylation on the diene bonds of Adda, and (3) internal interactions on the cyclic structure of MC-LR.

**Conclusion:**

Ag_3_PO_4_ is a highly efficient catalyst for MC-LR degradation in aqueous solutions.

## Introduction

Eutrophication promoted by human activities causes the proliferation of *cyanobacteria* or blue–green algae [1]. *Microcystis* species are the most prevalent *Cyanobacteria* worldwide; these species produce hepatotoxin microcystins (MCs), a possible carcinogen. More than 90 types of MCs have been identified to date [2], [3]. As a representative congener, microcystin-LR (MC-LR) is a common toxic species in contaminated aquatic systems and persists for long periods.

The general structure of the MCs is cyclo (*D* -Ala-*L*-X-*D*-erythro-*β*-Methyl-*D*-isoAsp-*L*-Y-Adda-isoGlu-N-methyldehydro-Ala), where X and Y represent variable amino acids and Adda represents 3-amino-9-methoxy-2,6,8-trimethyl-10-phenyldeca-4,6-dienoic-acid [4]. MCs are stable in natural aquatic systems because of their cyclic structure. The degradation of MC-LR is always slow by physical or biological methods. Therefore, developing a method for the fast removal of MC-LR is a significant research work.

Semiconductor materials used as environment-friendly photocatalysts for the removal of hazardous organic contaminants have drawn considerable attention over the last few decades. TiO_2_-based heterogeneous photocatalytic oxidation leads to water splitting [5]–[7]. Methods that promote pollutant mineralization are based on the generation of highly reactive oxygen species (e.g., HO•, O•_2_^−^). Nevertheless, the photons in the visible region that occupy a major portion of the solar spectrum cannot be utilized by TiO_2_ because of its large band gap (*E*_g_ = 3.2 eV), which reduces the degradation effect and increases the catalyst amount [6], [8]. Yi et al. have recently reported that silver orthophosphate (Ag_3_PO_4_) was a highly efficient water oxidation visible light photocatalyst that could harness visible light to oxidize water and the oxygen evolution rate with Ag_3_PO_4_ was 2.5 times that with BiVO_4_ [9], [10]. Furthermore, many studies have also shown that Ag_3_PO_4_ has relatively good performance for the degradation of organic pollutants under visible light irradiation [11]. However, the degradation capacity of Ag_3_PO_4_ for MC-LR under visible light irradiation has yet to be reported.

This study aims to investigate the degradation capacity of Ag_3_PO_4_ for MC-LR under visible light. The influencing parameters, including initial pH, initial Ag_3_PO_4_ concentration, initial MC-LR concentration, and recycle experiments, were studied. The degradation intermediates of MC-LR were also examined by liquid chromatography/mass spectrometry (LC/MS), and the degradation mechanism was analyzed.

## Materials and Methods

### Chemical and reagent

MC-LR standard sample was purchased from Express Technology Co., Ltd. (China) and kept at −20°C. The MC-LR agent used for removal was isolated and purified from cultured FACHB905 (Institute of Hydrobiology, Chinese Academy of Sciences, Wuhan, China) by using an improved method proposed by Ma et al. [12]. The purity of the MC-LR agent was over 95%, as determined by high-performance liquid chromatography (HPLC, Agilent 1200). AgNO_3_ (99%) was purchased from Snn Chemical and Technology Co., Ltd. (Shanghai, China). Analytical-grade Na_2_HPO_4_•12H_2_O, NaOH, and H_3_PO_4_ were purchased from Shanghai Chemical Reagent Co., Ltd. (China). Acetonitrile (HPLC grade) and trifluoroacetic acid (TFA, 99.5+%, HPLC grade) were purchased from Alfa Aesar, USA.

### Synthesis and characteristics of Ag_3_PO_4_

Ag_3_PO_4_ was synthesized by the ion-exchange method as follows. AgNO_3_ powder (0.075 mol) was dissolved in 500 mL of deionized H_2_O. Then, 0.1 mol/L of Na_2_HPO_4_ (250 mL) aqueous solution was added in drops into the above solution under continuous stirring until yellow precipitates were formed. Finally, the mixture was washed with distilled water and dried at 60°C in air overnight.

The crystallographic properties of the products were obtained by X-ray diffraction (XRD, PANalytical/X' Pert PRO, Cu Kα radiation, Holland), *λ* = 1.54056 Å. The surface morphology and particle size of the photocatalyst were examined by a field-emission scanning electron microscope (SEM, Philips XL30, Holland). The ultraviolet-visible diffuse reflectance spectra were recorded with a UV-vis spectrophotometer using BaSO_4_ as the reference (PerkinElmer, Lambda 35, USA).

### Photocatalytic degradation of MC-LR

The effects of parameters such as initial pH, initial Ag_3_PO_4_ concentration, initial MC-LR concentration, and recycle experiments on the degradation capacity of Ag_3_PO_4_ for MC-LR were evaluated. The different initial pH values were 3.19, 5.01, 6.74, 8.76, and 11.96, by using 0.1 mol/L NaOH or 0.1 mol/L H_3_PO_4_ as buffer. The different initial Ag_3_PO_4_ concentrations used were 0, 7.07, 13.67, 20.40, 26.67, and 33.47 g/L. MC-LR solution was extracted from cultured algal cells. A 20.34 mg/L MC-LR stock solution was diluted with distilled water to obtain the concentration gradient (2.61, 4.05, 6.57, 8.30, 9.06, 9.76, and 10.09 mg/L). The stability of the as-prepared Ag_3_PO_4_ photocatalyst was investigated by four successive cycling runs of experiments with pH 5.06, and the catalyst amount and the initial MC-LR concentration used in each run were 26.67 and 9.06 mg/L, respectively. After 5 h of visible light irradiation, the suspension was extracted from the beaker and centrifuged at 5000 rpm for 20 min to remove the impurities. The solid catalyst was washed thrice with deionized water and then further centrifuged. Finally, the solid catalyst was dried at 60 °C for 12 h for the next run [13]. Moderate MC-LR solution was added into a 15 mL quartz glass vial, in which Ag_3_PO_4_ was added. A preferable test interval of 1 h was adopted to further analyze the degradation process, with each initial parameter having three parallel samples.

Photocatalytic degradation was conducted at 26±1°C and atmospheric pressure under visible light irradiation. The mixture was magnetically stirred in the dark for 60 min to ensure the adsorption−desorption equilibrium of MC-LR on the catalysts and the adsorption of approximately 5% MC-LR. The reactor was then irradiated with visible light emitted by a 500 W xenon lamp with a 420 nm cutoff filter. A sample of 0.8 mL was taken and filtered to remove the photocatalyst particles. The quantification of MC-LR in each sample was determined by HPLC. For HPLC analysis, the injection volume to a C-18 discovery column (Supelco) was 20 µL at 40 °C. The mobile phase in isocratic mode with a flow rate of 1 mL/min was a mixture of 40% acetonitrile and 60% water, both containing 0.05% TFA. MC-LR was measured with a photodiode array detector at 238 nm.

### LC-MS Analysis

A Bruker micrOTOF II mass spectrometer was utilized for the MS identification of the reaction intermediates. Positive ions were analyzed using electrospray ionization, and MS data were acquired in full-scan mode (400 Da/scan to 1200 Da/scan). The voltage of electrospray process was 4 kv. The MS consisted of a Dionex Ultimate 3000 HPLC Pump and a Dionex Ultimate 3000 autosampler. The HPLC column was ZORBAX SB-C18 column (4.6 mm×250 mm, 5 µm particle size, Agilent, USA), and the temperature was maintained at 40 °C. The mobile phase consisted of a mixture of 0.05% TFA in acetonitrile and 0.1% TFA in Milli-Q water. Gradient elution method was programmed according to the study of Liu et al. [14]. The flow rate was controlled at 1.0 mL/min. The injection volume of the treated sample was 20 µL. A 1 mg standard of MC-LR dry powder was prepared by adding 20 mL Milli-Q water.

### Degradation kinetic analysis

As widely used of the kinetic model in the scope of chemical reaction or photo catalytic degradation process [13], [15], [16], the influence of each factor on MC-LR degradation was described by the pseudo-first-order kinetic model (Equation 1):
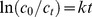
(1)


Where 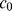 and 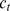 were the concentrations of reactant at time 0 and *t*, respectively, and 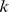 was the pseudo-first-order rate constant. 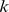 was obtained from the gradient of the graph of 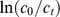 versus 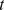.

## Results

### Characteristics of the Ag_3_PO_4_ photocatalyst

**Figure 1:**
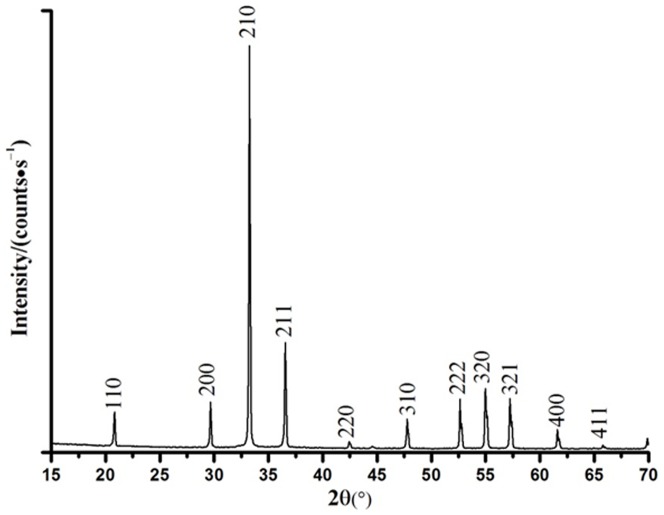
XRD patterns of the Ag_3_PO_4_ powders.

The powder was subjected to XRD to determine the crystal phase composition and crystallite size of Ag_3_PO_4_. As shown in Figure 1, all diffraction peaks of the as-prepared Ag_3_PO_4_ samples were in line with the standard spectrum (JCPDS card No. 06-0505). In addition, no diffraction peaks resulting from impurities were detected [17]. The eleven distinctive peaks at 20.83°, 29.66°, 33.27°, 36.55°, 42.47°, 47.78°, 52.68°, 54.98°, 57.24°, 61.60°, and 65.87° matched with the (110), (200), (210), (211), (220), (310), (222), (320), (321), (400), and (411) crystal planes of Ag_3_PO_4_, respectively.

**Figure 2:**
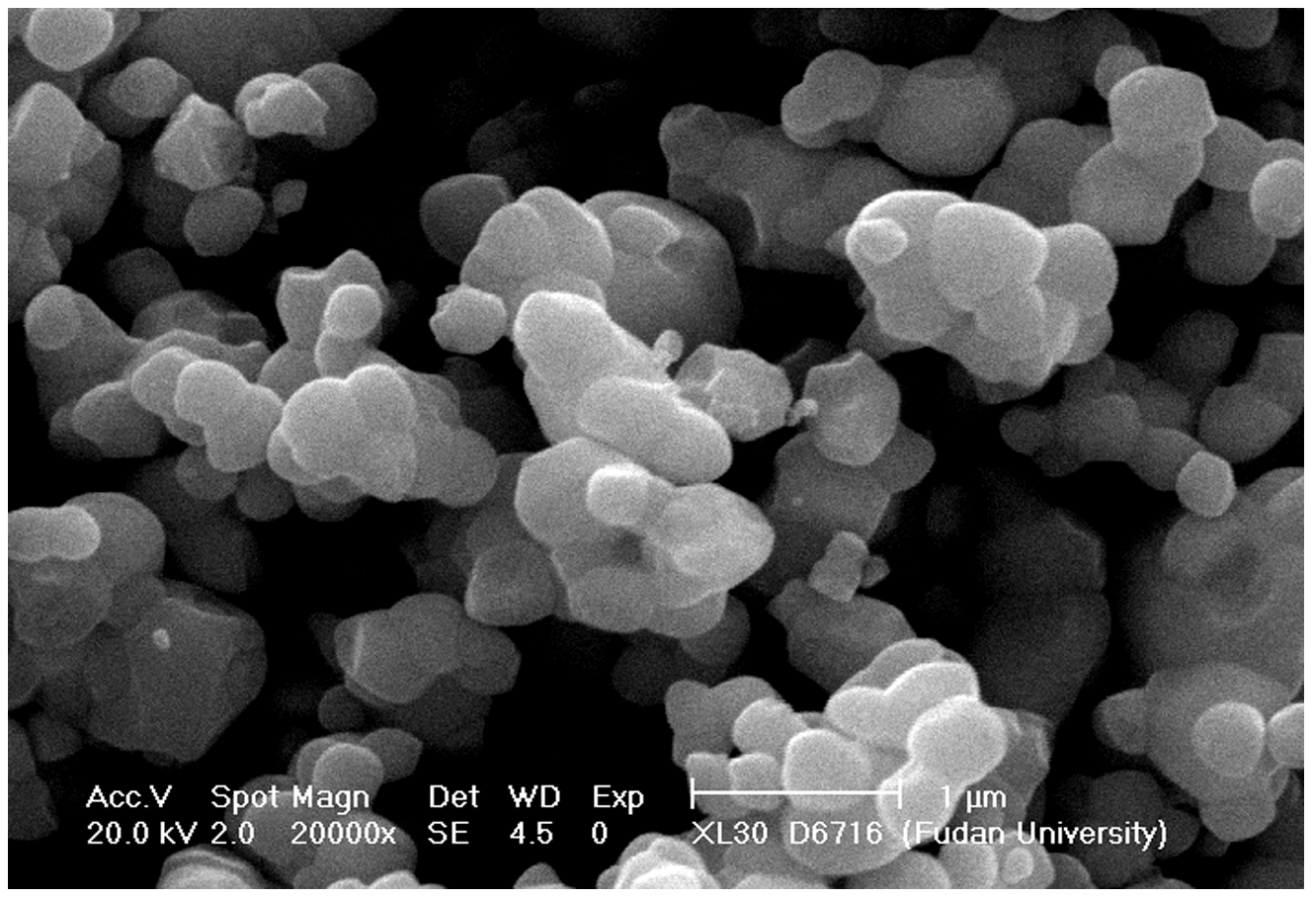
SEM image of the as-synthesized Ag_3_PO_4_ photocatalyst.

The morphologies and particle of the as-synthesized Ag_3_PO_4_ were demonstrated in the SEM images. As shown in Figure 2, Ag_3_PO_4_ consisted of agglomerated smooth spherical particles with an average particle size of approximately 200 nm to 500 nm.

**Figure 3:**
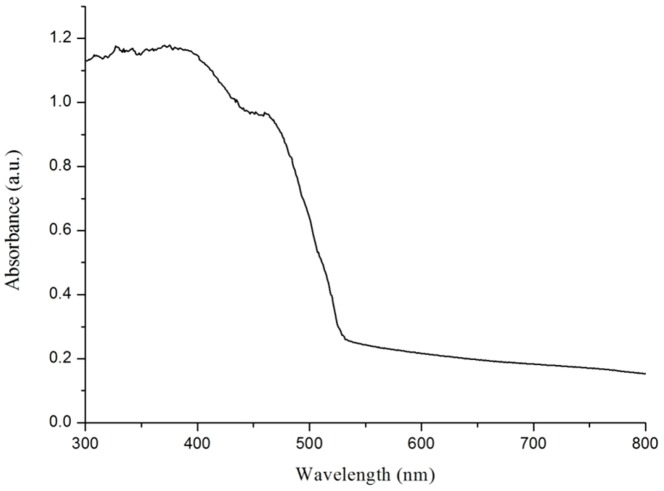
UV−vis diffuse reflectance spectra of the as-synthesized Ag_3_PO_4_ photocatalyst.

The UV–vis diffuse reflectance spectra showed the optical properties of the Ag_3_PO_4_ photocatalyst. As shown in Figure 3, the absorption band edge of the prepared Ag_3_PO_4_ was at about 530 nm in the spectrum. The optical absorption near the band edge of a crystal obeys the following Equation 2 [18]:
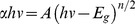
(2)


Where 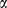, 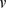, and 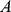 were absorption coefficient, light frequency, and proportionality constant, respectively; and 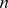 decided the transition type in a semiconductor (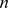 = 1, direct absorption; 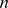 = 4, indirect absorption; 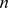 for Ag_3_PO_4_ was 1) [13]. By applying this equation, the band gap of Ag_3_PO_4_ was approximated as 2.35 eV, which was significantly lower than that of TiO_2_.

### Degradation characterization of MC-LR using Ag_3_PO_4_

#### Effect of initial pH

**Figure 4:**
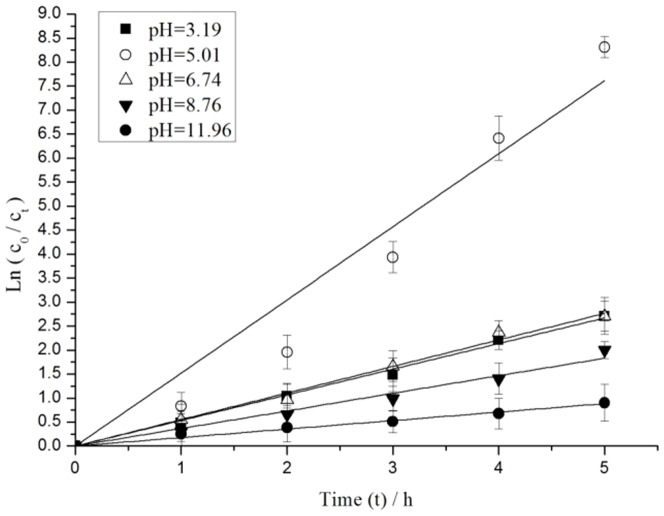
Pseudo-first-order kinetics of MC-LR degradation under different initial pH values.

Figure 4 shows the degradation profile of MC-LR under visible light irradiation at different initial pH with the same initial Ag_3_PO_4_ concentration of 26.67 g/L and initial MC-LR concentration of 9.06 mg/L. The maximum MC-LR degradation rate occurred at pH 5.01, with the pseudo-first-order kinetic constant *k* of 1.52 h^−1^ and a MC-LR removal rate of 99.98% in 5 h. The degradation rate of MC-LR increased as the pH was increased from 3.19 to 5.01. By contrast, the degradation rate of MC-LR reduced when the pH was higher than 5.01. The pseudo-first-order kinetic constant *k* dropped to 0.18 h^−1^ and only 59.19% MC-LR was removed when the pH was 11.96.

The photocatalytic degradation of MCs was pH dependent [19]. Photocatalytic oxidation occurred on or near the surface of the catalyst in the Ag_3_PO_4_ catalysts system. The change in pH influenced hydrophobicity of MC-LR, which was increased as pH decreases. Through the hydrophobic effect, hydrophobic compounds preferentially moved to surfaces from the bulk of aqueous solvents [19]. The MC-LR showed very low absorption under basic conditions (pH 8.76 and 11.96). These findings demonstrated that hydrophobicity was a contributory factor influencing the initial adsorption of MC-LR to the surface of Ag_3_PO_4_. The change in pH also influenced solubility and catalytic activity of Ag_3_PO_4_ catalysts. On one hand, Ag_3_PO_4_ catalysts could be dissolved in the strong acidic solution which would reduce the catalyst dosage and then led the decrease of absorption [18]. On the other hand, the concentration of hydroxide ion (OH^−^) might affect catalytic activity of Ag_3_PO_4_ in aqueous solution. In the basic solution, excess OH^−^ would promote the formation of Ag^+^ to metallic Ag. Ag deposited onto the surface of Ag_3_PO_4_ particles in the form of Ag nanoparticles to cover the active sites, which influenced the absorption of visible light [18]. The results suggested that Ag_3_PO_4_ catalysts exhibited the best photocatalytic performance for MC-LR degradation at pH 5.01.

#### Effect of initial Ag_3_PO_4_ concentration

**Figure 5:**
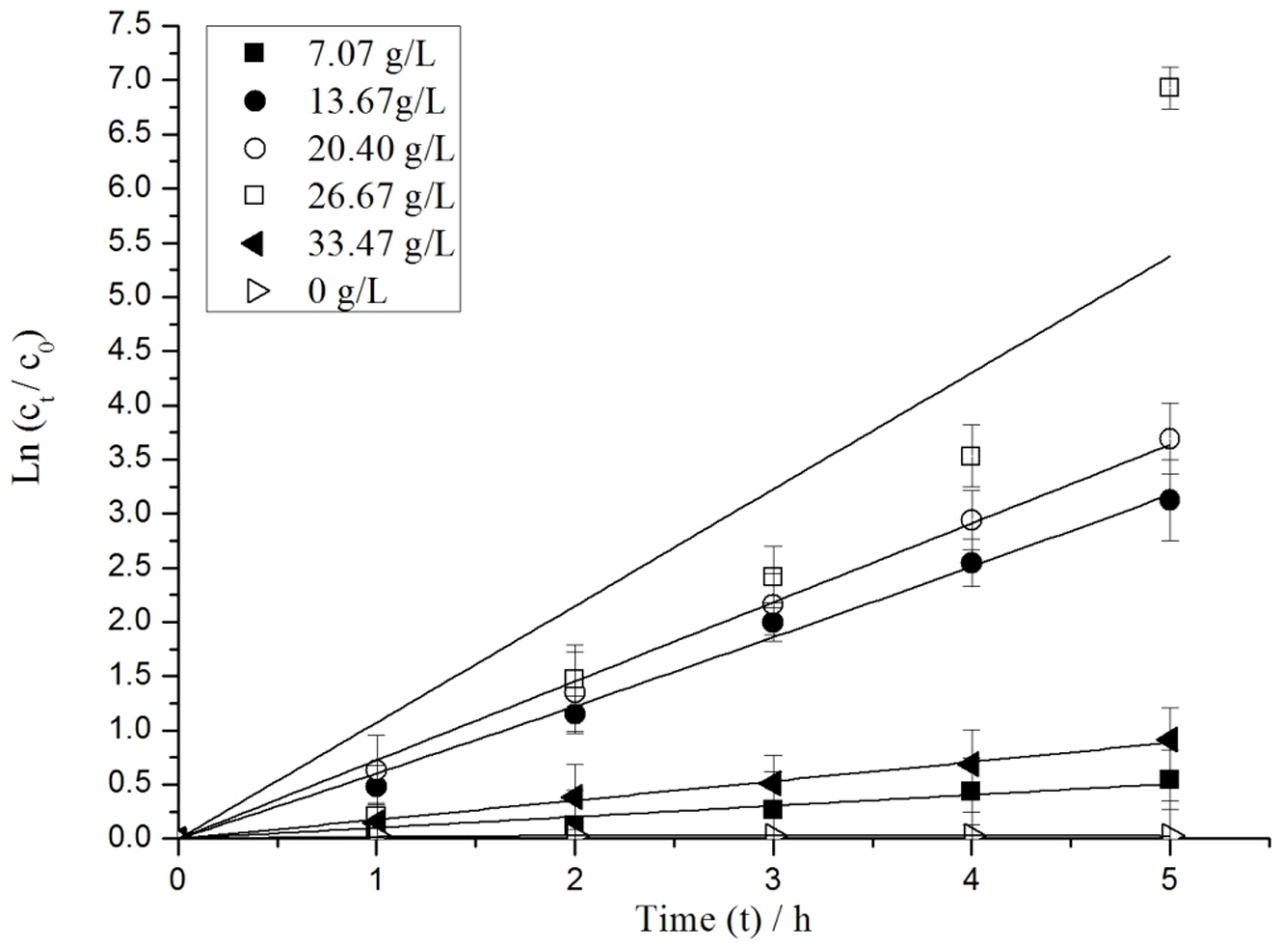
Pseudo-first-order kinetics of MC-LR degradation under different initial Ag_3_PO_4_ concentration.

Figure 5 shows the degradation of MC-LR (*c*_0_ = 9.76 mg/L) for various initial concentrations of Ag_3_PO_4_ at pH 5.06. The control experiment showed that the removal of MC-LR without Ag_3_PO_4_ under visible light irradiation was almost negligible. The removal rate for MC-LR increased from 41.90% to 99.90% when the initial Ag_3_PO_4_ concentration was increased from 7.07 g/L to 26.67 g/L. The pseudo-first-order kinetic constant *k* reached 1.08 h^−1^ when the Ag_3_PO_4_ concentration was 26.67 g/L. Nevertheless, the degradation rate of MC-LR was reduced to 59.91% when the Ag_3_PO_4_ concentration was increased to 33.47 g/L. Therefore, the optimal concentration of Ag_3_PO_4_ was 26.67 g/L. A maximum value of the effective area of receiving light was detected in the photocatalytic system. The photocatalytic system reached a saturation condition as the initial concentration of Ag_3_PO_4_ was further increased from 26.67 g/L to 33.47 g/L. Excessive dosage of catalyst led to the scattering effect, which affected the light absorption of the photocatalyst and then decreased the pseudo-first-order kinetic constant [20]. Ag_3_PO_4_ displayed a high photocatalytic activity under weak acidic condition (k = 1.08 h^−1^). The photocatalytic activity of Ag_3_PO_4_ was 0.8 times that of mesoporous N-TiO_2_ (k = 0.60 h^−1^), the well-known TiO_2_ system photocatalyst with the best photocatalytic performance [21].

#### Effect of initial MC-LR concentration

**Figure 6:**
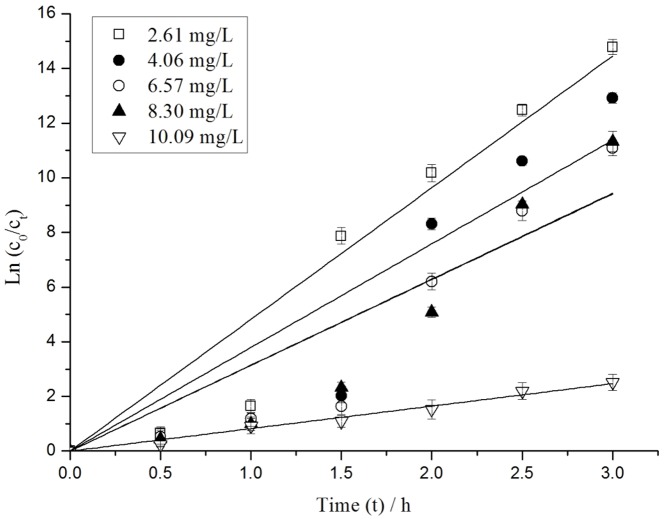
Pseudo-first-order kinetics of MC-LR degradation under different initial MC-LR concentration.

Figure 6 shows the degradation rate at different initial MC-LR concentrations when the initial Ag_3_PO_4_ concentration was 26.67 g/L, initial pH was approximately 5.07, and the test interval was 0.5 h. The degradation of MC-LR was significantly influenced by the concentration of MC-LR. When the initial MC-LR concentration was increased from 2.61 mg/L to 10.09 mg/L, the degradation rate of MC-LR decreased from nearly 100% to 91.92% and the pseudo-first-order kinetic constant *k* decreased from 4.82 h^−1^ to 0.82 h^−1^ after 3 h of reaction. These results indicated that the degradation efficiency was decreasing with the MC-LR concentration. The yield rate of HO• was remained as a constant with the same Ag_3_PO_4_ concentration. The increasing concentration of MC-LR resulted in a decrease in the ratio of decomposed amount of MC-LR to the total amount of MC-LR.

#### Effect of recycle experiments of Ag_3_PO_4_ photocatalyst

**Figure 7:**
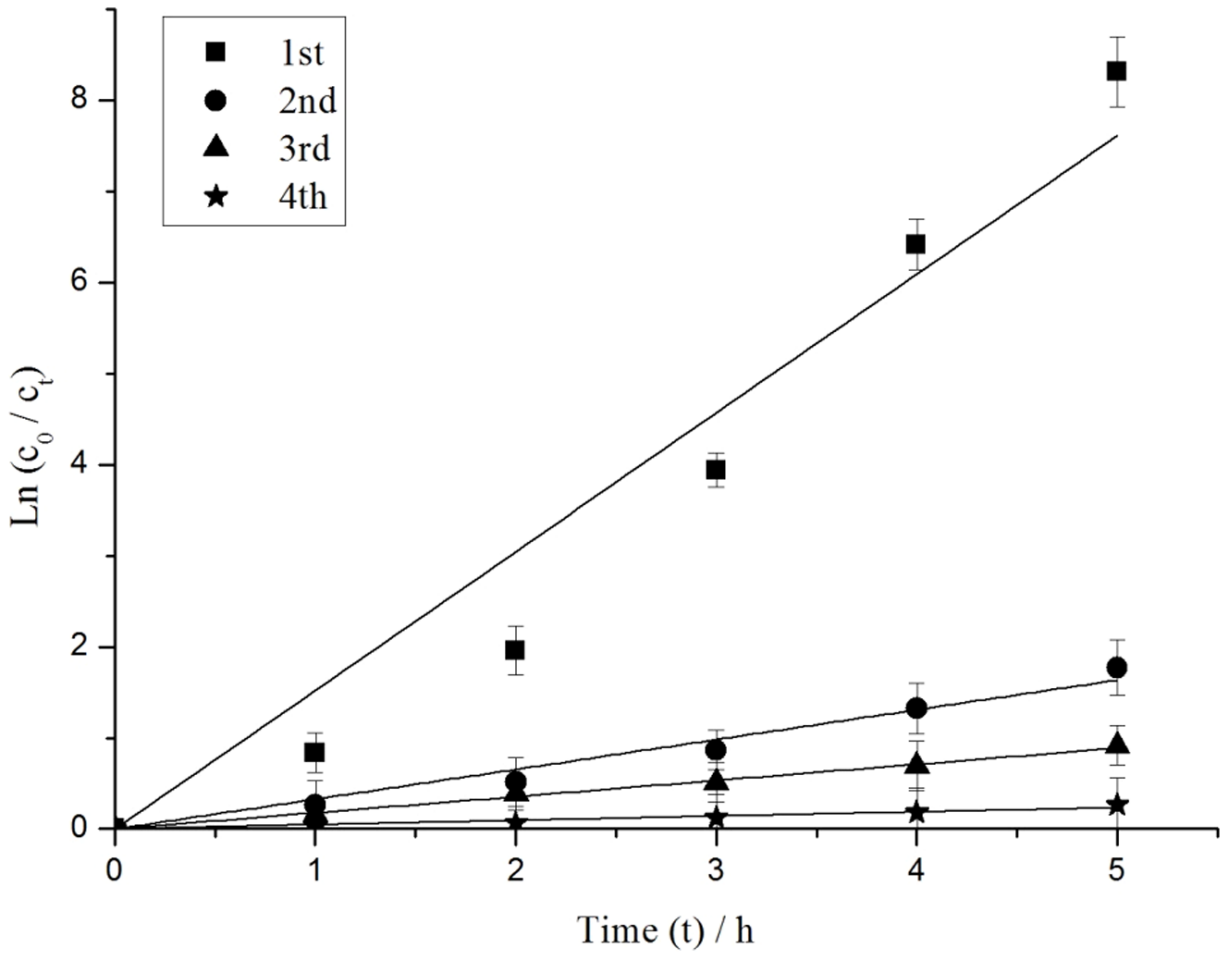
Pseudo-first-order kinetics of MC-LR degradation under 4 cycling runs.

As shown in Figure 7, the degradation rate of MC-LR gradually reduced with increasing cycling runs of Ag_3_PO_4_. The pseudo-first-order kinetic constant *k* decreased from 1.52 h^−1^ to 0.05 h^−1^ when the cycling runs of Ag_3_PO_4_ were increased from 1 to 4. The photocatalytic activity of the Ag_3_PO_4_ product had a significantly loss. Possibly, the increased number of cycling runs promoted the deposition of the Ag nanoparticles on the surfaces of the Ag_3_PO_4_ particles during the photocatalytic process. The deposited nanoparticles covered the active sites [22]. Hence, Ag_3_PO_4_ exhibited the best photocatalytic performance for MC-LR degradation in the first run.

Error caused by logarithmic computation and automatic integration of peak area made some data were not well fitted the pseudo-first-order kinetic model. Nevertheless, parallel experiment analyzed the error of data was within 10%, which revealed that the data were reliable.

Therefore, the degradation parameters including initial pH, initial Ag_3_PO_4_ concentration, initial MC-LR concentration, and recycle experiments were in line with the pseudo-first-order kinetic model. All factors are listed in Table 1. The maximum MC-LR degradation rate of 99.98% can be obtained within 5 h under the following optimum conditions: pH of 5.01, Ag_3_PO_4_ concentration of 26.67 g/L, MC-LR concentration of 9.06 mg/L, and cycling run of one time.

**Table 1:** Degradations kinetics of MC-LR in different conditions.

Factors	Conditions	Pseudo-first-order kinetics ln (*c_0_*/*c_t_*) = *kt*	
		*k* (h^−1^)	*R^2^*
pH	3.19	0.53	0.99813
	5.01	1.52	0.97555
	6.74	0.55	0.99649
	8.76	0.36	0.99100
	11.96	0.18	0.99435
Ag_3_PO_4_ (g/L)	0.00	0.01	0.79063
	7.07	0.10	0.97653
	13.67	0.63	0.99735
	20.40	0.73	0.99895
	26.67	1.08	0.91456
	33.47	0.18	0.99769
MC-LR (mg/L)	2.61	4.82	0.9696
	4.06	3.79	0.91146
	6.57	3.15	0.91345
	8.30	3.14	0.91289
	10.09	0.82	0.99248
cycling runs	1^st^ Run	1.52	0.97555
	2^nd^ Run	0.33	0.98872
	3^rd^ Run	0.11	0.99254
	4^th^ Run	0.05	0.97268

### Degradation mechanisms of MC-LR

Photocatalytic degradation depends on the electron–hole pairs that are produced from the inter-band excitation. When the Ag_3_PO_4_ photocatalyst absorbs radiation with energy equal to or larger than its band gap, the electrons and holes that migrate to the surface of catalyst serve as redox sources and react with adsorbed water to generate highly reactive oxygen species (e.g., HO•). According to previously described degradation mechanisms with HO•, this reactive oxygen species can change the structure of MC-LR or trigger some internal reactions on the cyclic structure of MC-LR [23]. The production of HO• by Ag_3_PO_4_ photocatalyst was shown in the following Equations 3–6:
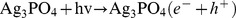
(3)

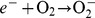
(4)

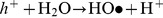
(5)

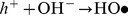
(6)


Where 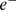 represents electrons in the conduction band and 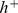 represents holes in the valence band.

The degradation products of MC-LR were observed in the total ion chromatogram of the LC/MS spectrogram. The peaks in the initial sample must have a signal-to-noise ratio of 3 and the difference between the peak areas of the initial and treated samples must be at least two times to eliminate the interference of impurities [5]. Ultimately, nine intermediates fitted these requirements: *m*/*z* 1011.5, 1027.5, 1029.5, 781.3, 835.4, 1009.5, 877.4, 764.3, and 681.4. The following three main degradation pathways were proposed according to the molecular weight of the products and the reaction mechanism between HO• and peptides or proteins.

#### Hydroxylation on the aromatic ring of Adda

The intermediates *m*/*z* 1011.5 with retention time (R.T.) = 20.7 min and 27.9 min probably belonged to the aromatic ring of Adda [24]. The radical HO• combined with the double bonds of the aromatic ring, formed a carbon-centered radical, and then formed a peroxy radical by reacting with oxygen [25]. The alkyl group directly connected to the aromatic ring of Adda donated the electrons. Electrophilic substitution with HO• radicals was believed to occur at the *ortho* and *para* positions.

**Figure 8:**
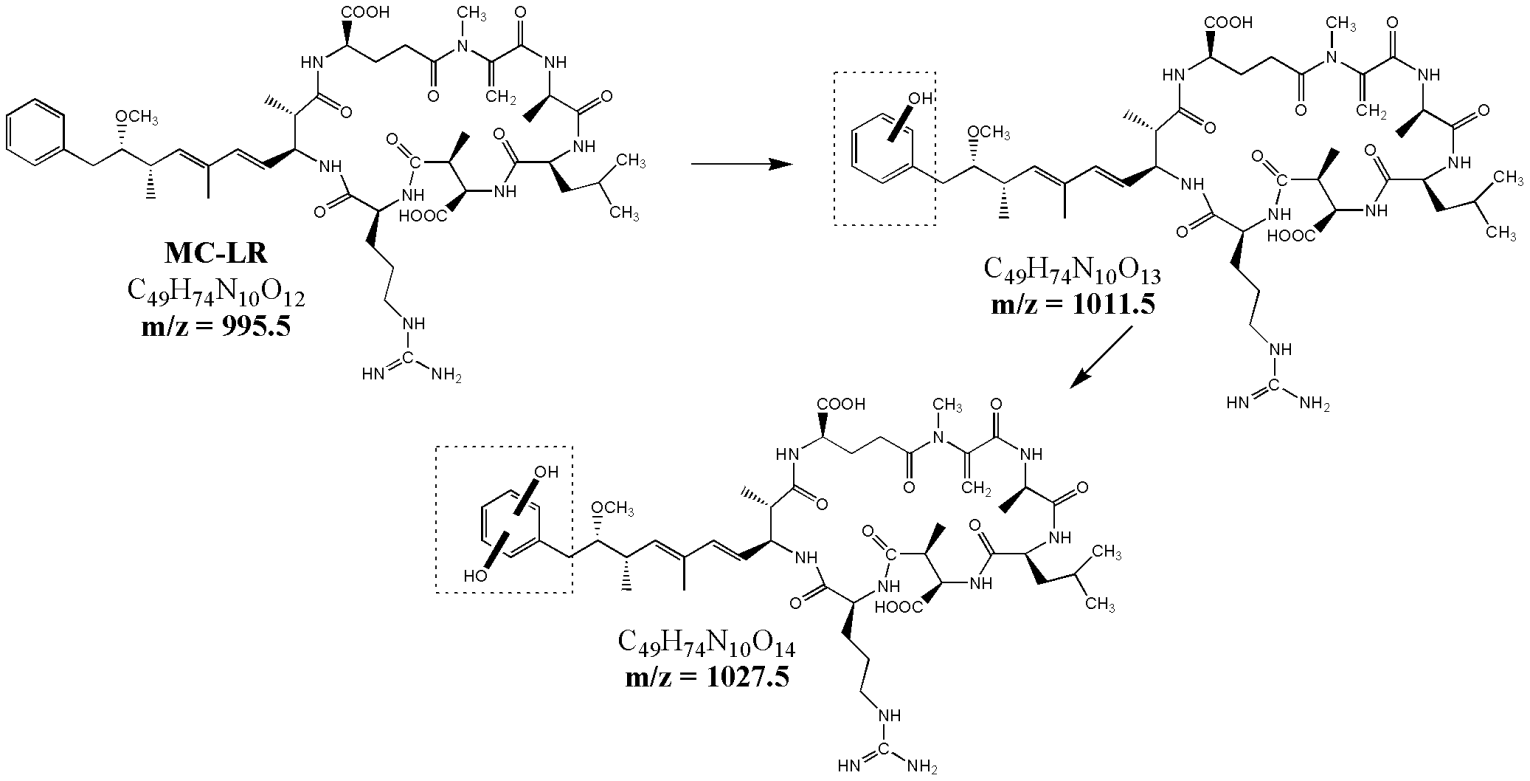
Proposed MC-LR's degradation pathways: hydroxylation on the aromatic ring of Adda.

The first aromatic hydroxylation increased the electron density on the aromatic ring and induced the second hydroxylation, which was *m*/*z* 1027.5 (Figure 8).

#### Hydroxylation on the diene bonds of Adda

**Figure 9:**
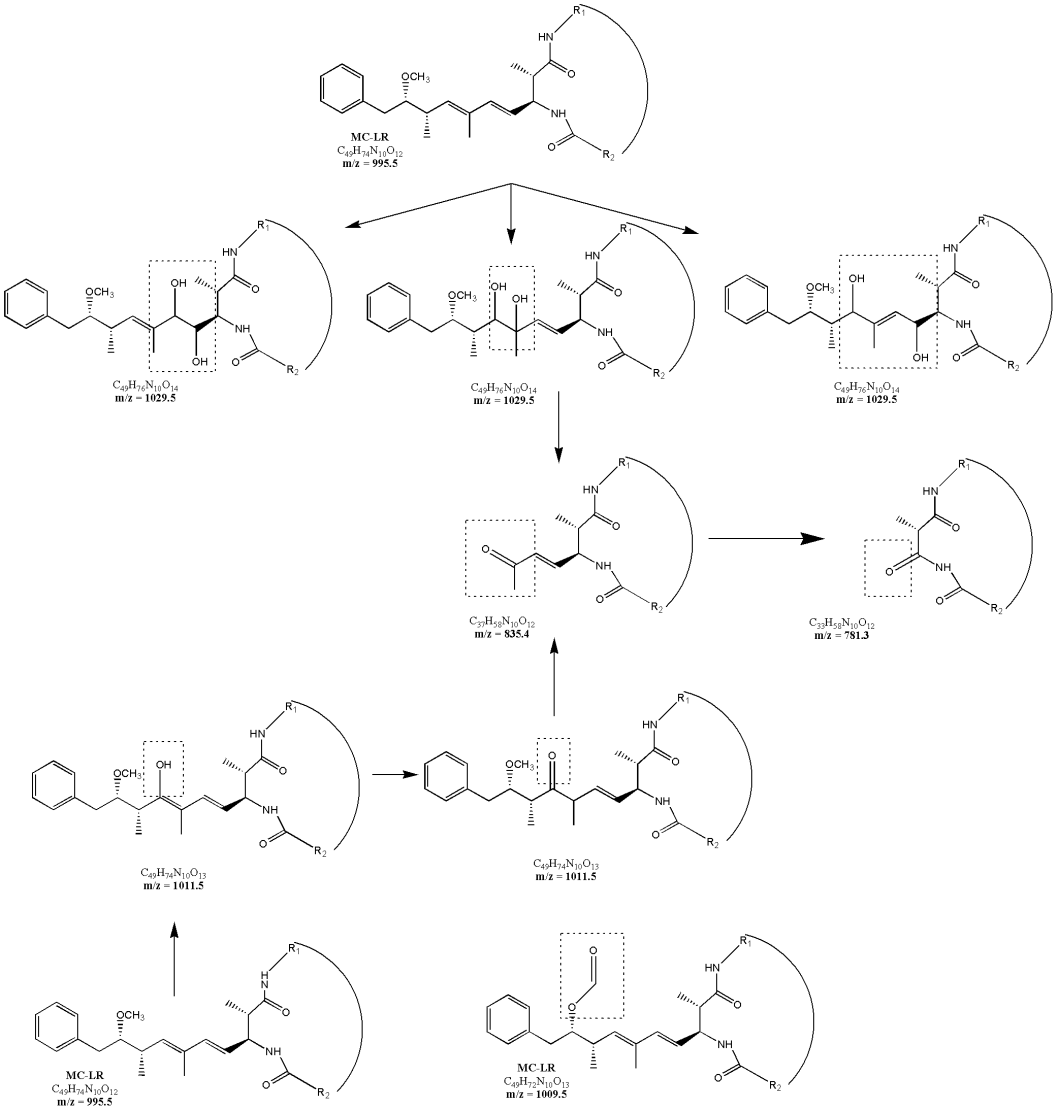
Proposed MC-LR's degradation pathways: hydroxylation on the diene bonds of Adda.

The diene bonds of the Adda chain was likely another attacked site. Hydroxyl addition possibly occurred between C_4_–C_5_, C_6_–C_7_, or to C_4_ and C_7_, forming the *m*/*z* 1029.5 intermediates (Figure 9) [25]. In the second oxidation route, the Adda chain was completely removed and formed the ketone-derivative *m*/*z* 781.3, which had never been reported yet. One probable pathway was the double hydroxyl addition to the C_6_−C_7_ of the Adda chain; this mechanism formed the *m*/*z* 1029.5 intermediate products. Then, the C_6_−C_7_ of the Adda chain was split to form the intermediate *m*/*z* 835.4. The intermediate *m*/*z* 835.4 was subjected to a series of oxidation processes, forming the *m*/*z* 781.3 ketone derivative with an intact cyclic structure. Another possible route was through the substitution of an HO group to the hydrogen of C_7_ of the Adda chain. This mechanism formed an enol-MC-LR (*m*/*z* 1011.5) that was isomerized to the ketone–MC-LR [25]. After a series of oxidative-induced bond cleavage steps, the ketone derivative *m*/*z* 781.3 was formed. The ketone derivative was more stable than hydroxyl derivative.

The detected *m*/*z* 1009.5 was probably formed by the hydrogen abstraction reaction on the methoxy group of Adda by HO•.

#### Internal interactions on the cyclic structure of MC-LR

**Figure 10:**
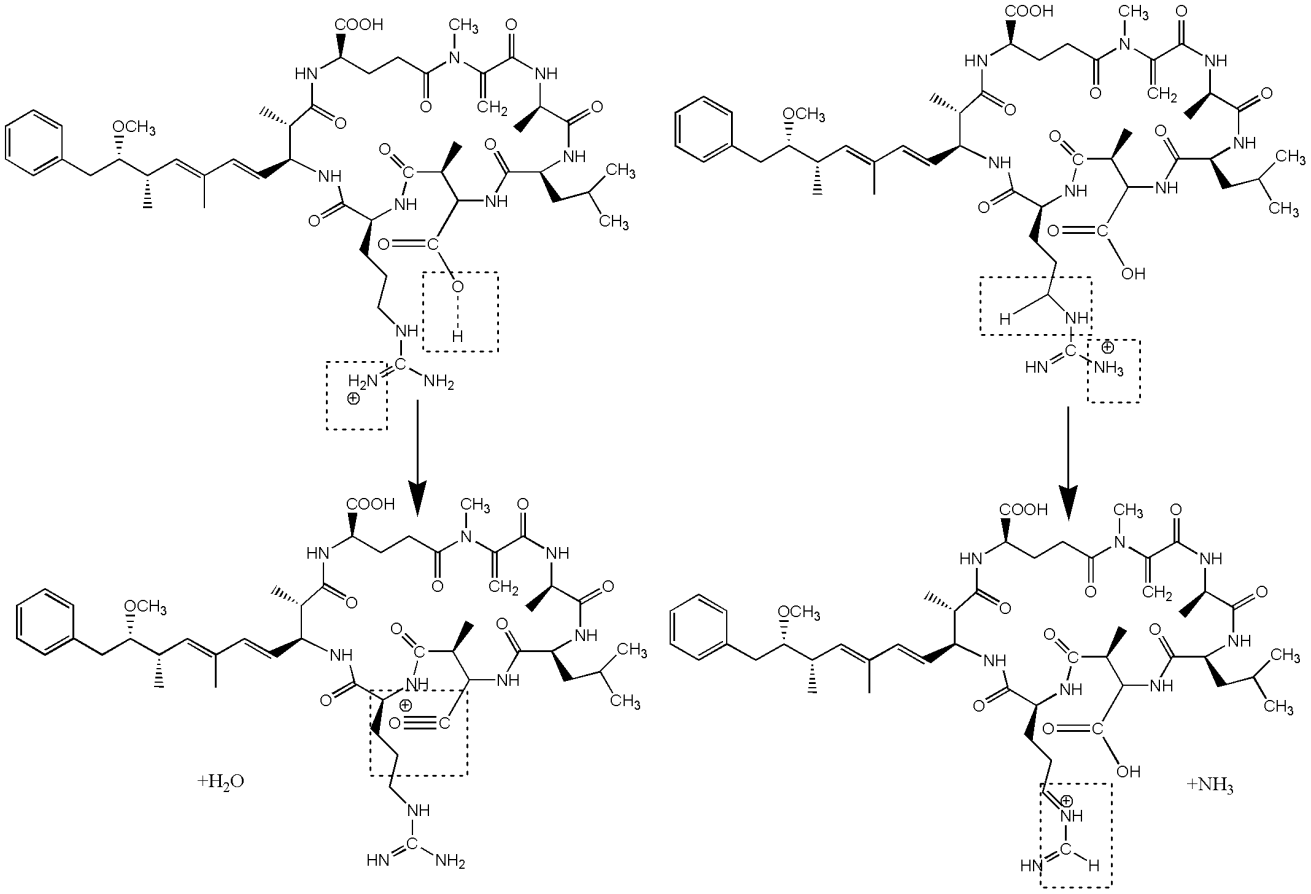
Possible interactions between Arg and MeAsp on the cyclic structure of MC-LR.

In the photocatalytic process, the excited Ag_3_PO_4_ photocatalyst that produced a large amount of HO• could form a strong oxidizing condition. This condition could trigger internal reactions on the cyclic structure of MC-LR; meanwhile, some neutral molecules (such as H_2_O, NH_3_, CO_2_, CO, and C_9_H_10_N) would be lost [26]. Dehydration occurred on the free carboxylic groups of amino acids, hydroxyl-substituted bonds, and the backbone of the peptides, which could form the linearization structure of MC-LR [6]. Studies showed that the dissociation energy of amino acids adjacent to Arg is evidently higher than those without Arg. Other works reported that the loss of an ammonia molecule lead to the cyclic structure open in non-Arg MC derivatives [27], [28]. In the case of MC-LR, MeAsp with a free carboxylic group adjoined to Arg was more likely to interact with the guanidine group of Arg than the peptide bond, followed by the loss of neutral molecules (Figure 10).

**Figure 11:**
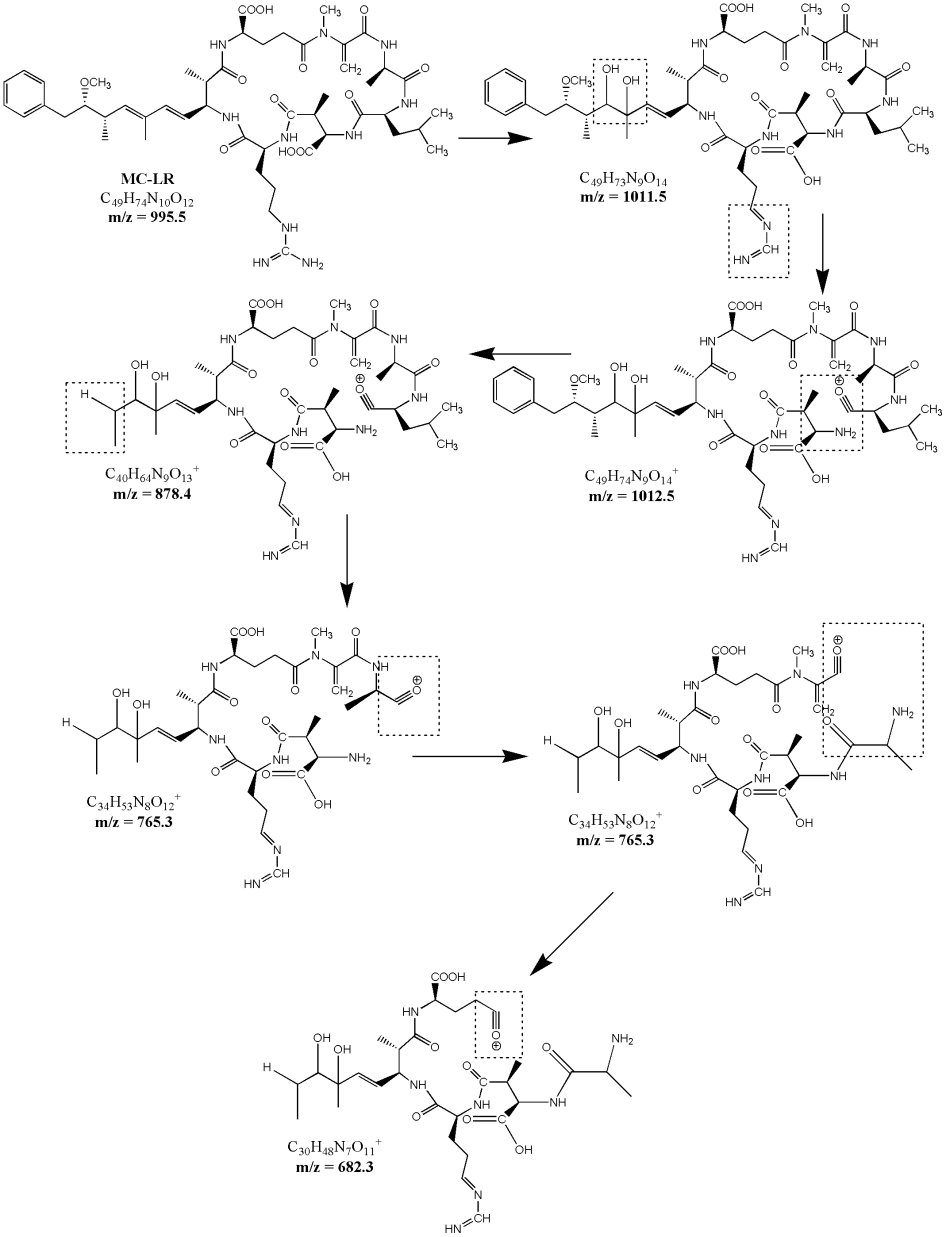
Proposed MC-LR's degradation pathways: internal interactions on the cyclic structure of MC-LR.

As shown in Figure 10, another intermediate *m*/*z* 1011.5 probably occurred because of the removal of ammonia from the Arg moiety and the double hydroxylation on the C_6_−C_7_ of the Adda chain. The peptide bond between MeAsp and Leu was fractured because Arg transferred to the nitrogen of MeAsp. Then, the Adda moiety was broken to form *m*/*z* 877.4. Then, the peptide bond between Leu and Mdha was fractured to form *m*/*z* 764.3. Ultimately, *m*/*z* 681.4 was formed by the removal of Mdha through the same mechanism. The pathways were in accordance with the study of Antoniou et al. [6] (Figure 11).

The diene bonds of Mdha were other sites for the double hydroxylation and oxidation of aldehydes, forming the *m*/*z* 1029.5 and 1011.5 fragments, respectively [5].

## Discussion

The Ag_3_PO_4_ photocatalyst was synthesized and used to remove MC-LR from aqueous solutions for the first time in this study. The degradation efficiency of MC-LR was influenced by several parameters, including initial pH, initial Ag_3_PO_4_ concentration, initial MC-LR concentration, and recycle experiments. In addition, the degradation process was well fitted with the pseudo-first-order kinetic model. Three main degradation pathways were proposed based on the molecular weight of the intermediates and the reaction mechanism: (1) hydroxylation on the aromatic ring of Adda, (2) hydroxylation on the diene bonds of Adda, and (3) internal interactions on the cyclic structure of MC-LR.

In conclusion, Ag_3_PO_4_ is a highly efficient catalyst for MC-LR degradation in aqueous solutions. However, further studies must be conducted to determine the entire degradation process. Furthermore, the property of the intermediates formed during the MC-LR degradation should be investigated to promote the practical application of the Ag_3_PO_4_ photocatalyst.
